# Morphological and molecular identification of cardiopulmonary nematodes in the European wildcat (*Felis silvestris*) and nine other carnivores in Spain

**DOI:** 10.1007/s00436-025-08612-y

**Published:** 2025-12-26

**Authors:** Javier Millán, Javier Marco, Ruth Rodríguez-Pastor, Fermín Urra, Juan Antonio Castillo, Diego Villanúa, María Paz Peris

**Affiliations:** 1 Instituto Agroalimentario de Aragón-IA2 (Universidad de Zaragoza-CITA), Miguel Servet 177, Zaragoza, 50013 Spain; 2 Fundación ARAID, Av. Ranillas 1-D, Zaragoza, 50018 Spain; 3 One Health Institute, Facultad de Ciencias de la Vida, Universidad Andres Bello, Av. República 252, Santiago, Chile; 4 Departamento de Patología Animal, Facultad de Veterinaria, Universidad de Zaragoza, Miguel Servet 177, Zaragoza, 50013 Spain; 5 Navarra Environmental Management (Orekan), Padre Adoain 219 Bajo, Pamplona, 31015 Spain

**Keywords:** Bronchopulmonary, Carnivoran, Helminth, Lungworm, Nematoda

## Abstract

Cardiopulmonary nematodes (CPN) are among the most pathogenic helminths, with wild carnivores serving as hosts for a variety of species. Knowledge of their presence in the Iberian Peninsula is limited, and for certain hosts, such as the European wildcat (*Felis silvestris*), no data are currently available. Lung and heart samples from 112 road-killed individuals belonging to 10 different species, primarily wildcats (*n* = 33), collected in Navarra (northern Spain) were collected. Morphological identification of nematodes was done and confirmed, when possible, by sequencing fragments of the cytochrome *c* oxidase subunit I (*cox1*) and the internal transcribed spacer 2 (*ITS2*) genes. Fifteen wildcats (45%) were found to be parasitized, with the following species identified: *Troglostrongylus brevior* (30%), *Angiostrongylus chabaudi* (15%), and *Oslerus rostratus* (3%). Other CPN-positive hosts included the Eurasian badger (*Meles meles*) (3/15), with *Angiostrongylus daskalovi* and *Crenosoma melesi*; the pine marten (*Martes martes*) (3/13) and the stone marten (*Martes foina*) (5/11), both parasitized by *Eucoleus aerophilus* and *Crenosoma petrowi*; and the European polecat (*Mustela putorius*) (4/8), with *Crenosoma melesi*. This study reports the first identification of *A. chabaudi* in the Iberian Peninsula and confirms previous, though rare, records of other CPN species. Additionally, several novel genetic sequences are provided for poorly characterized taxa. Conducting parasitological surveys in free-roaming domestic cats in areas where wildcats are present, to assess the potential for cross-species transmission of these parasites, is highly recommended.

## Highlights

*Troglostrongylus brevior*, *Angiostrongylus chabaudi*, and *Oslerus rostratus* identified in wildcats.

*Angiostrongylus daskalovi*, *Eucoleus aerophilus*,* Crenosoma melesi* and *Crenosoma petrowi* identified in mustelids.

All American and European minks, weasels, otters and genets were negative.

New *cox1* and *ITS2* sequences are provided for poorly characterized parasites.

## Introduction

Cardiopulmonary nematodes (CPNs) parasitize the vascular and respiratory systems of both wild and domestic animals (Traversa et al. 2010, 2021; Brianti et al. 2013), causing a variety of respiratory symptoms, including nasal discharge, tachypnea, and, in more severe cases, dyspnea (Urquhart et al. 2001; Brianti et al. 2013). The heartworm can also lead to serious and potentially fatal conditions such as thromboembolism, proliferative arteritis, pulmonary hypertension, pulmonary edema, and *cor pulmonale*, ultimately resulting in heart failure in dogs (Venco 2007). CPNs have also been implicated in pathological processes affecting wild carnivores (Stevanović et al. 2018; Penezić et al. 2022; Mechouk et al. 2024) and their role in respiratory disease among neonatal animals has been demonstrated (Brianti et al. 2013).

In Europe, *Oslerus osleri* and *Aelurostrongylus abstrusus* have been reported in domestic and wild carnivores (Gerrikagoitia et al. 2010; Traversa et al. 2010; 2021; García-Livia et al. 2023). *Oslerus rostratus* has also increasing interest due to new data on its geographic distribution in domestic cats (Millán and Casanova 2009; Jefferies et al. 2010; Brianti et al. 2014; Varcasia et al. 2015; Kiszely et al. 2019; García-Livia et al. 2023) and European wildcats (*Felis silvestris*) (Diakou et al. 2021). The occurrence of *Troglostrongylus brevior* may also be rising in both wild and domestic felids (Brianti et al. 2014). *Filaroides martis* has been reported in various mustelids (Torres et al. 2008; Monakhov 2023) and *Eucoleus aerophilus* is relatively common in wild and domestic carnivores (Ryšavá-Nováková 2009; Traversa and Di Cesare 2013; Samorek-Pieróg et al. 2023). *Perostrongylus falciformis* has been identified in Eurasian badgers (*Meles meles*) across several European regions (Torres et al. 2001; Deak et al. 2018; Byrne et al. 2019). The genus *Angiostrongylus* is also widespread in European wildlife. *Angiostrongylus vasorum* is a well-known pathogen of domestic dogs (Helm et al. 2010) and its incidence appears to be rising in wild canids as well (Germitsch et al. 2020; Taylor et al. 2015). *Angiostrongylus chabaudi* parasitizes the right ventricle of the heart and the pulmonary arteries of wildcats in Italy, Greece, and Romania (Varcasia et al. 2014, 2015; Diakou et al. 2016, 2021; Veronesi et al. 2023), while *A. daskalovi* has been identified in badgers (Gerrikagoitia et al. 2010; Gherman et al. 2016a; Nagy et al. 2021), as well as in invasive American mink (*Neogale vison*) (Martínez-Rondán et al. 2017). Members of the genus *Crenosoma* are broadly distributed across Europe (Deak et al. 2023), parasitizing a wide range of hosts including wild and domestic canids (Latrofa et al. 2015; Deak et al. 2023), mustelids (Torres et al. 2001; Deak et al. 2023; Latrofa et al. 2015), and ursids (Addison and Fraser 1994). *Crenosoma melesi* and *Crenosoma petrowi* are the most common species in mustelids and ursids in Europe (Addison and Fraser 1994; Deak et al. 2023), whereas *Crenosoma vulpis* primarily parasitizes canids (Latrofa et al. 2015). Notably, many of these parasites frequently co-occur, with co-infection rates approaching 25% in some wildcat populations (Annoscia et al. 2014; Falsone et al. 2014; Traversa et al. 2015).

*Dirofilaria immitis* is the only heart-dwelling helminth (Matsuda et al. 2003; Penezić et al. 2018; Genchi et al. 2019; Ionică et al. 2022; Markakis et al. 2024). Its increasing importance stems from to the severe health problems in the typical host (domestic dogs), the emergence of macrocyclic lactone resistance and the withdrawal from the market in Europe of melarsomine (Traversa et al. 2024). Although the domestic dogs is its main host (Genchi et al. 2019), this nematode can parasitize a wide range of wild carnivores (Matsuda et al. 2003; Markakis et al. 2024; Varcasia et al. 2015; Penezić et al. 2018; Ionică et al. 2022; Veronesi et al. 2023).

Knowledge of CPNs in Iberian carnivores remains geographically patchy and taxonomically limited, mostly restricted to species parasitizing some mustelids, viverrids, and canids (Alvarez et al. 1991; Feliú et al. 1996; Torres et al. 2001, 2003; Millán et al. 2004; Segovia et al. 2007; Martínez-Carrasco et al. 2007; Gerrikagoitia et al. 2010; Martínez-Rondán et al. 2017, 2019). To date, no studies have focused on the European wildcat in the Iberian Peninsula. Wild carnivores are phylogenetically close to domestic dogs and cats and are believed to share most, if not all, helminth species. Moreover, the feeding ecology of carnivorans—often involving possible intermediate hosts of CPNs such as amphibians, reptiles, birds, and rodents—further increases their exposure to parasitic infection. It is therefore essential to identify the species involved, along with their prevalence and distribution. In this study, the rich carnivore biodiversity of Navarra, a Spanish autonomous region, is capitalized to investigate the occurrence of cardiopulmonary nematodes in Iberian wild carnivores, with particular emphasis on the European wildcat and the European mink (*Mustela lutreola*)—two menaced species for which no previous data are available from this region.

## Materials and methods

### Specimen collection

Carcasses of road-killed animals were collected by Environmental Agents of the Government of Navarra, who recorded the location point, and transported to the Wildlife Recovery Center in Ilundáin. There, the specimens were stored frozen until necropsy. During necropsy, the animals were weighted and measured, and the heart and lungs were extracted, placed in self-sealing bags, and refrozen prior to further analysis. All samples were subsequently processed at the Parasitology Laboratory of the Department of Pathology, Faculty of Veterinary Medicine, University of Zaragoza. In total, 112 individuals representing ten species from the families Mustelidae, Viverridae, and Felidae were examined (Table 1).

**Table 1:** Sample size for the diverse carnivora species included in the study, number of parasitized individuals and cardio-pulmonary helminth species retreived

Common name	Scientific name	*n*	Total positive	Species retrieved	Positive for each taxon
Family Mustelidae					
Eurasian badger	*Meles meles*	15	3	*Angiostrongylus daskalovi* *Crenosoma melesi*	2 1
Pine marten	*Martes martes*	13	3^a^	*Crenosoma petrowi* ^b^ *Eucoleus aerophilus* ^b^	2 3
Stone marten	*Martes foina*	11	5^a^	*Crenosoma petrowi* ^b^ *Eucoleus aerophilus* ^b^	1 5
American mink	*Neogale vison*	10	0		
European mink	*Mustela lutreola*	5	0		
European polecat	*Mustela putorius*	8	4	*Crenosoma melesi*	4
Least weasel	*Mustela nivalis*	3	0		
Eurasian otter	*Lutra lutra*	4	0		
Family Viverridae					
Common genet	*Genetta genetta*	9	0		
Family Felidae					
European wildcat	*Felis silvestris*	33	15^a^	*Angiostrongylus chabaudi* *Oslerus rostratus* *Troglostrongylus brevior*	5 1 10

^a^ Some individuals were parasitized by more than one helminth species

^b^ Morphological identifications not confirmed by molecular means

### Nematode collection and morphological identification

Nematodes were extracted using two dissection protocols. The first protocol, applied to the heart, consisted of opening the atria and ventricles with scissors. The internal contents were then examined under a stereomicroscope. Following initial observation, the heart chambers were rinsed with tap water, and the washings were collected for further analysis. The resulting fluid was transferred to Falcon-type tubes and subjected to sedimentation for 40 min. After decanting the supernatant, the sediment was examined under a stereomicroscope to detect the presence of nematodes (Lemming et al. 2020). Bronchopulmonary helminths were extracted by dissecting the trachea and bronchial tree, followed by examination of the internal surfaces under a stereomicroscope. To recover additional parasites, the lungs were submerged in containers filled with potable water, allowing visible adult nematodes to be collected using forceps. The resulting washing fluid was then subjected to a sedimentation process in Falcon-type tubes for 40 min to isolate any remaining helminths. To maximize the recovery of parasites from lung tissue, distal fragments of the pulmonary lobes were subjected to enzymatic digestion using a solution of pepsin (activity 1:10,000) and 1.5% hydrochloric acid (HCl) in 100 mL of distilled water. The digestion process was carried out at 40 °C for one hour with gentle agitation. The resulting material was filtered through a 63 μm mesh sieve to isolate nematodes (Martínez-Rondán et al. 2017). All adult helminths, larvae, and eggs recovered were preserved in Eppendorf-type tubes containing 90% ethanol. Morphological identification was performed based on measurements obtained under a stereomicroscope using a micrometer, after clearing the specimens with lactophenol. Diagnostic features and morphometric data were referenced from standard taxonomic manuals (Anderson 2000) and relevant scientific literature (Feliú et al. 1996; Costa et al. 2003; Traversa et al. 2008, 2015; Gerrikagoitia et al. 2010; Brianti et al. 2014; Gherman et al. 2016b; Deak et al. 2023).

### Molecular techniques and phylogenetic analysis

The initial objective was to obtain at least one sequence per parasite and host species for a fragment of the mitochondrial cytochrome *c* oxidase subunit I gene (*cox1*; Protocol #1), as well as for the second internal transcribed spacer region (*ITS2*), using previously published polymerase chain reaction (PCR) protocols specific to each target (Table 2). For *Crenosoma* specimens, due to the absence of reference sequences for the suspected species in these gene regions, a different *cox1* fragment was amplified and sequenced (Protocol #2).

**Table 2:** Primers used for molecular confirmation of species identification

Gene	Primer name	Primer sequence	Length	Reference
*cox1* Protocol #1	JB3-Fw	5’-TTTTTTGGGCATCCTGAGGTTTAT- 3’	∼400 bp	(Bowles et al. 1993; Millán and Blasco-Costa 2012)
	JB4-Rv	5’-TAAAGAAAGAACATAATGAAAATG- 3’		
*cox1* Protocol #2	LCO-Fw	5’- GGTCAACAAATCATAAAGATATTGG-3’	∼700 bp	(Caldeira et al. 2003; Folmer et al. 1994)
	HCO-Rv	5’-TAAACTTCAGGGTGACCAAAAAATCA- 3’		
*ITS2*	NC1-Fw	5’- ACGTCTGGTTCAGGGTTGTT-3’	∼500 bp	(Caldeira et al. 2003; Gasser et al. 1993)
	NC2-Rv	5’-TTAGTTTCTTTTCCTCCGCT-3’		

Genomic DNA was extracted from ethanol-preserved nematode samples using the Speedtools DNA Extraction Kit (Biotools, B & M Labs, S.A., Madrid, Spain). Prior to extraction, samples were rinsed with physiological saline to eliminate ethanol residues. Subsequently, 200 µL of lysis buffer and 25 µL of Proteinase K were added, and nematode tissues were homogenized using a sterile lancet. The samples were incubated at 70 °C for 1 h. The entire lysate was transferred to extraction columns, and the manufacturer’s instructions were followed for the remaining steps. Extracted DNA was eluted in 100 µL of elution buffer and stored at − 20 °C until further use.

Amplifications of the *cox1* and *ITS2* regions were carried out in a final reaction volume of 20 µL, containing 10 µL of N Supreme NZYTaq II DNA polymerase (NZYtech Lda, Lisbon, Portugal), 10 µM of each primer, 2.5 µL of template DNA, and DNase-free water. For *cox1* amplification, a touchdown PCR protocol was used: initial denaturation at 95 °C for 5 min, followed by 40 cycles of denaturation at 95 °C for 45 s, annealing at 50 °C for 1 min, and extension at 72 °C for 2 min, with a final extension at 72 °C for 5 min. For *ITS2* amplification, the PCR protocol consisted of an initial denaturation at 94 °C for 90 s, followed by 39 cycles of denaturation at 94 °C for 50 s, annealing at 58 °C for 1 min, and extension at 72 °C for 90 s, with a final extension at 72 °C for 10 min.

PCR products (15 µL) were submitted to STAB VIDA, Lda (Caparica, Portugal) for unidirectional Sanger sequencing, using 10 µM of the corresponding PCR primer, following the company’s specifications. Raw sequences were manually assembled and edited using BioEdit version 7.2. Sequence identity was assessed through the Basic Local Alignment Search Tool (BLAST) searches (https://blast.ncbi.nlm.nih.gov) against publicly available data in GenBank^©^. Multiple sequence alignment was performed using MUSCLE, and phylogenetic relationships were inferred using the Maximum Likelihood algorithm. The best-fit models for phylogenetic analysis were determined using the “Models” function in MEGA. All analyses were conducted with MEGA11: Molecular Evolutionary Genetics Analysis version 11 (Tamura et al. 2021).

### Statistical analysis

Potential differences in parasite occurrence across bioregions (Eurosiberian vs. Mediterranean) were assessed using Fisher’s exact test. Additional risk factor analyses were conducted exclusively for the European wildcat, as sample sizes for the other species were insufficient for statistical inference. Generalized Linear Models (GLMs) were used to evaluate the effect of sex and age on parasite presence in wildcats. A body condition index was calculated as the ratio of the cube of tarsus length to body weight. Differences in body condition were also analyzed using GLMs, with sex and age included as covariates. Statistical significance was set at *p* < 0.05. All analyses were performed using IBM SPSS Statistics, Version 26.0.

## Results

A total of 32 individuals tested positive for the presence of nematodes (Table 1). The only species with a sample size greater than 30, the European wildcat, showed a prevalence of 45.4% (15/33). Polyparasitism was detected in only three individuals—one pine marten (*Martes martes*), one stone marten (*Martes foina*), and one wildcat—with each hosting two distinct taxonomic groups. Due to the poor preservation status of many carcasses, parasite abundance and intensity data were not recorded, as in several cases only partial specimens were recovered.

Helminths belonging to six taxonomic groups were morphologically identified: *Angiostrongylus* spp., *O. rostratus*, *T. brevior*, *Crenosoma* spp., and *E. aerophilus*. Specimens of *Angiostrongylus* from wildcats were identified as *A. chabaudi*, while those from badgers were morphologically assigned to *A. daskalovi*. All *Crenosoma* specimens recovered from polecats and badgers were identified as *C. melesi*. Specimens of *Crenosoma* from martens were morphologically consistent with *C. petrowi*.

**Fig. 1:**
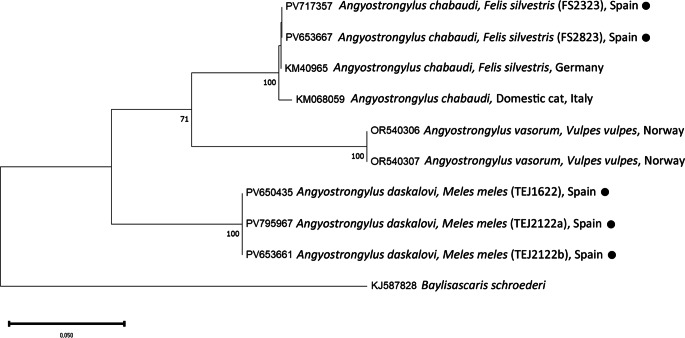
Phylogenetic analysis of the different *Angiostrongylus* sequences (*cox1*; Protocol #1) and other relevant reference sequences deposited in GenBank. The evolutionary model used was the Tamura Nei 93+ G. New sequences obtained in the present study are indicated with a black dot

**Fig. 2:**
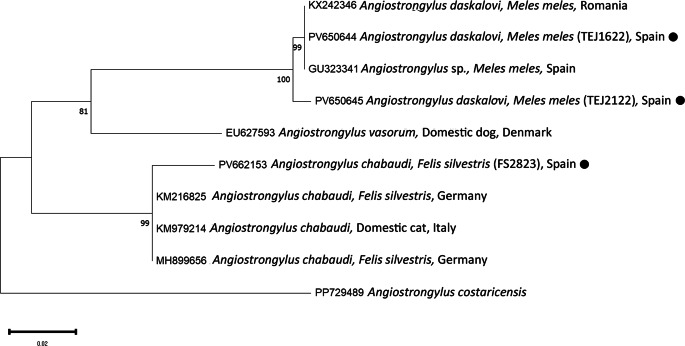
Phylogenetic analysis of the different *Angiostrongylus* sequences (*ITS2*) and other relevant reference sequences deposited in GenBank. The evolutionary model used was the Tamura Nei 92. New sequences obtained in the present study are indicated with a black dot

**Fig. 3:**
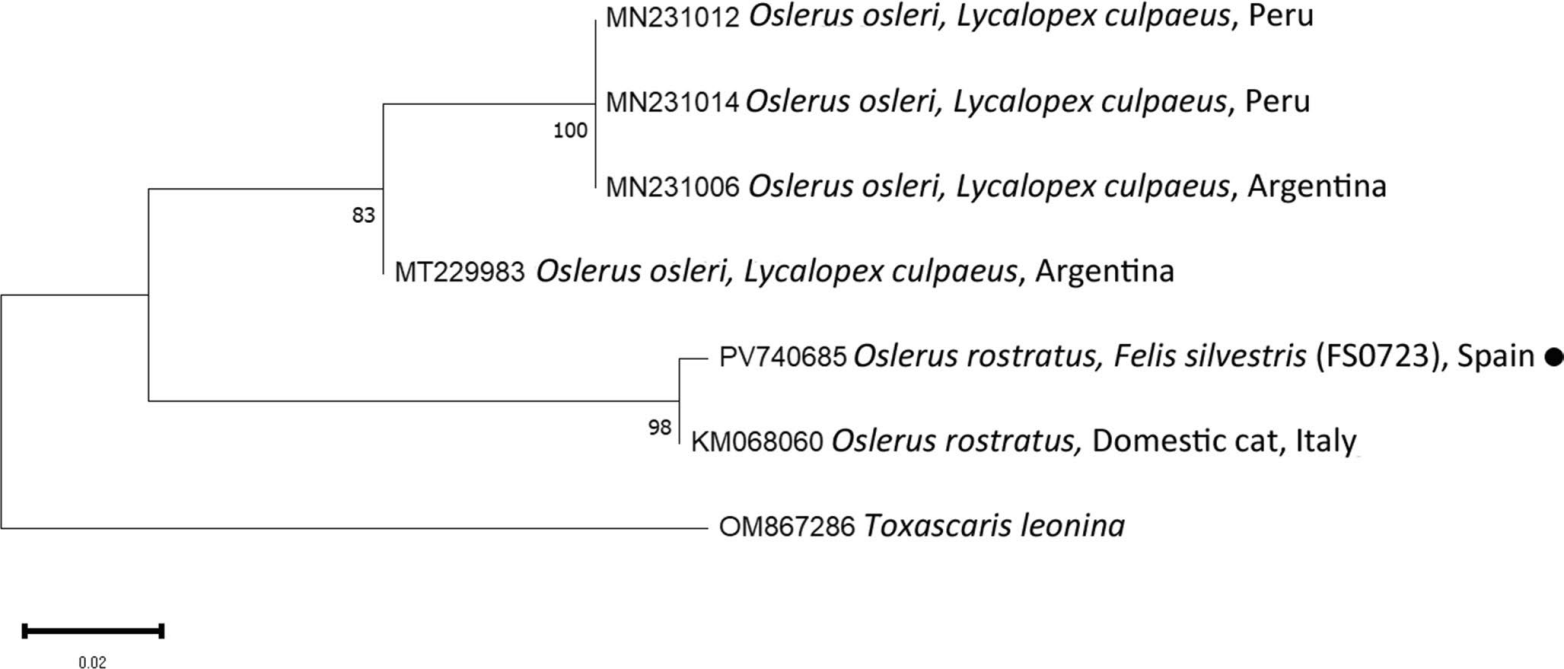
Phylogenetic analysis of the different *Osrelus* sequences (*cox1*; Protocol #1) and other relevant reference sequences deposited in GenBank. The evolutionary model used was the Hasegawa-Kishino-Yano + G. New sequences obtained in the present study are indicated with a black dot

**Fig. 4:**
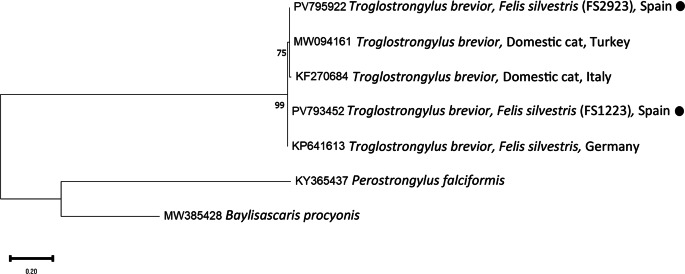
Phylogenetic analysis of the different *Troglostrongylus* sequences (*cox1*; Protocol #1) and other relevant reference sequences deposited in GenBank. The evolutionary model used was the Hasegawa-Kishino-Yano + G. New sequences obtained in the present study are indicated with a black dot

**Fig. 5:**
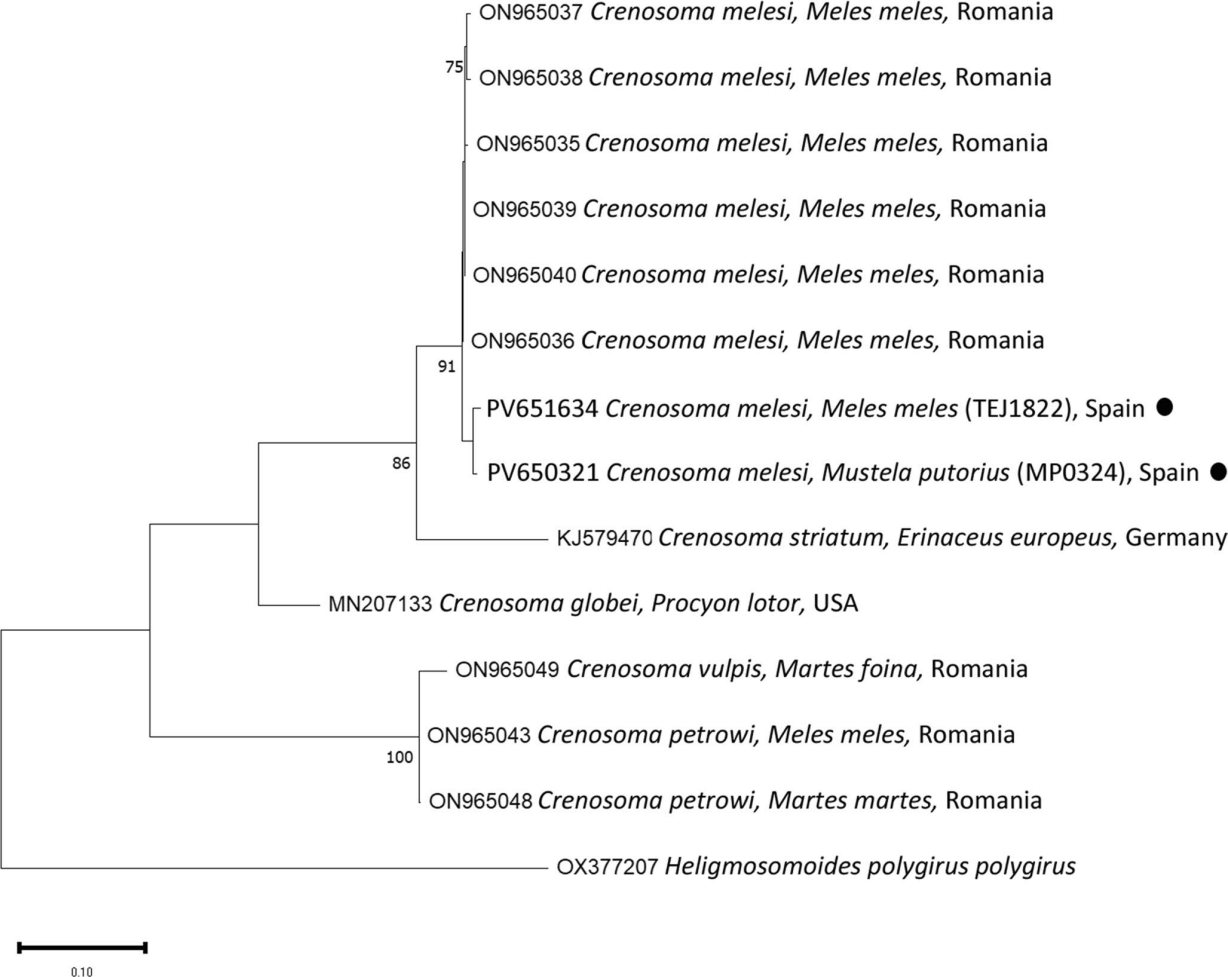
Phylogenetic analysis of the different *Crenosoma* sequences (*cox1*; Protocol #2) and other relevant reference sequences deposited in GenBank. The evolutionary model used was the Hasegawa-Kishino-Yano + G. New sequences obtained in the present study are indicated with a black dot

**Fig. 6:**
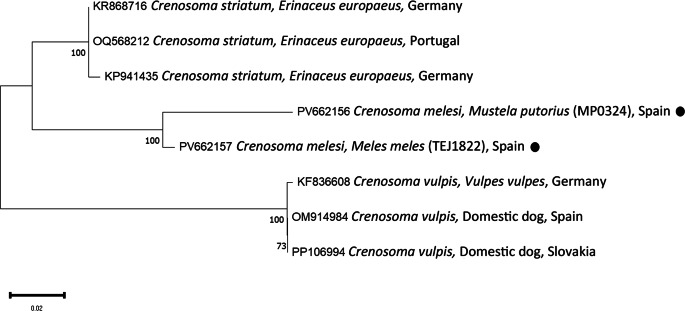
Phylogenetic analysis of the different *Crenosoma* sequences (*ITS2*) and other relevant reference sequences deposited in GenBank. The evolutionary model used was the Tamura Nei 92.New sequences obtained in the present study are indicated with a black dot

PCR and sequencing of selected individuals confirmed the morphological identifications. High-quality sequences of various gene fragments were obtained from eleven specimens (Tables 3 and 4). Additional sequences were of lower quality; however, BLAST analysis supported the morphological diagnoses. No readable sequences were obtained for *Eucoleus aerophilus* or for *Crenosoma* spp. infecting martens. Phylogenetic analyses corroborated the BLAST results (Figs. 1,2,3,4 and 5). For *C. melesi*, no *ITS2* reference sequences were available in GenBank. In the *ITS2* phylogenetic tree, our sequences clustered in a sister branch to *C. striatum* (Fig. 6), consistent with the placement observed in the *cox1*-based phylogeny generated using Protocol #2.

**Table 3:** Summary of readable *cox1* and *ITS2* sequences obtained from *Angiostrongylus* spp., *Oslerus rostratus* and *Troglostrongylus brevior* specimens from Iberian wild carnivores

		cox1 (protocol #1)					ITS2					
Host ID	Sp.	Accession number (this study)	Closest sequence	Query cover	Identity	Accession number	Accession number (this study)	Closest sequence	Query cover	Identity	Accession number	
*Angiostrongylus chabaudi*												
FS2823	Wildcat	PV653667	*A. chabaudi* isolate wk100	99%	99.51%	KM409651	PV662153	*A. chabaudi* isolate wk100	90%	98.33%	KM216825	
FS2323	Wildcat	PV717357	*A. chabaudi* isolate wk100	95%	99.75%	KM409651		-				
*Angiostrongylus daskalovi* ^a^												
TEJ1622	Badger	PV650435	*A. chabaudi* isolate wk100	99%	91.58%	KM409651	PV650644	*A. daskalovi* isolate RO	100%	100%	GU323341	
TEJ2122	Badger	PV795967	*A. chabaudi* isolate wk100	99%	90.91%	KM409651		-				
TEJ2122	Badger	PV653661	*A. chabaudi* isolate wk100	99%	91.62%	KM409651	PV650645	*A. daskalovi* isolate RO	100%	99.13%	GU323341	
*Oslerus rostratus*												
FS0723	Wildcat	PV740685	*O. rostratus* isolate 1	80%	99.70%	KM068060		-				
*Troglostrongylus brevior*												
FS1223	Wildcat	PV793452	*T. brevior* isolate Wk62	100%	99.73%	KP641613.1	PV662155	*T. brevior*	100%	100%	MH537789	
FS2923	Wildcat	PV795922	*T. brevior* isolate Wk62	99%	98.55%	KP641613.1	PV662154	*T. brevior* isolate Wk9	100%	97.35%	KM506759	
FS2723	Wildcat		-				PV650643	*T. brevior*	100%	99.02%	MH537789	

^a^ No *Angiostrongylus daskalovi cox1* gene sequences were available in GenBank

**Table 4:** Summary of readable *cox1* and *ITS2* sequences obtained from *Crenosoma melesi* from Iberian wild carnivores

MP0324-Polecat							TEJ1822-Badger				
Accession number (this study)	Closest sequence	Query cover	Identity			Accession number	Accession number (this study)	Closest sequence	Query cover	Identity	Accession number
***cox1*** **(protocol #1)**											
PV651633	*C. vulpis*	98%		88.80%	KM216824		PV796053	*C. vulpis*	100%	88.51%	KM216824
***cox1*** **(protocol #2)**											
PV650321	*C. melesi* isolate CJ007001	100%		98.75%	ON965035		PV651634	*C. melesi* isolate CJ007068	100%	98.75%	ON965036
***ITS2***											
PV662156	*C. striatum* clone K12	89%		90.75%	KR868715		PV662157	*C. striatum* clone K12	100%	93.96%	KR868715

**Fig. 7:**
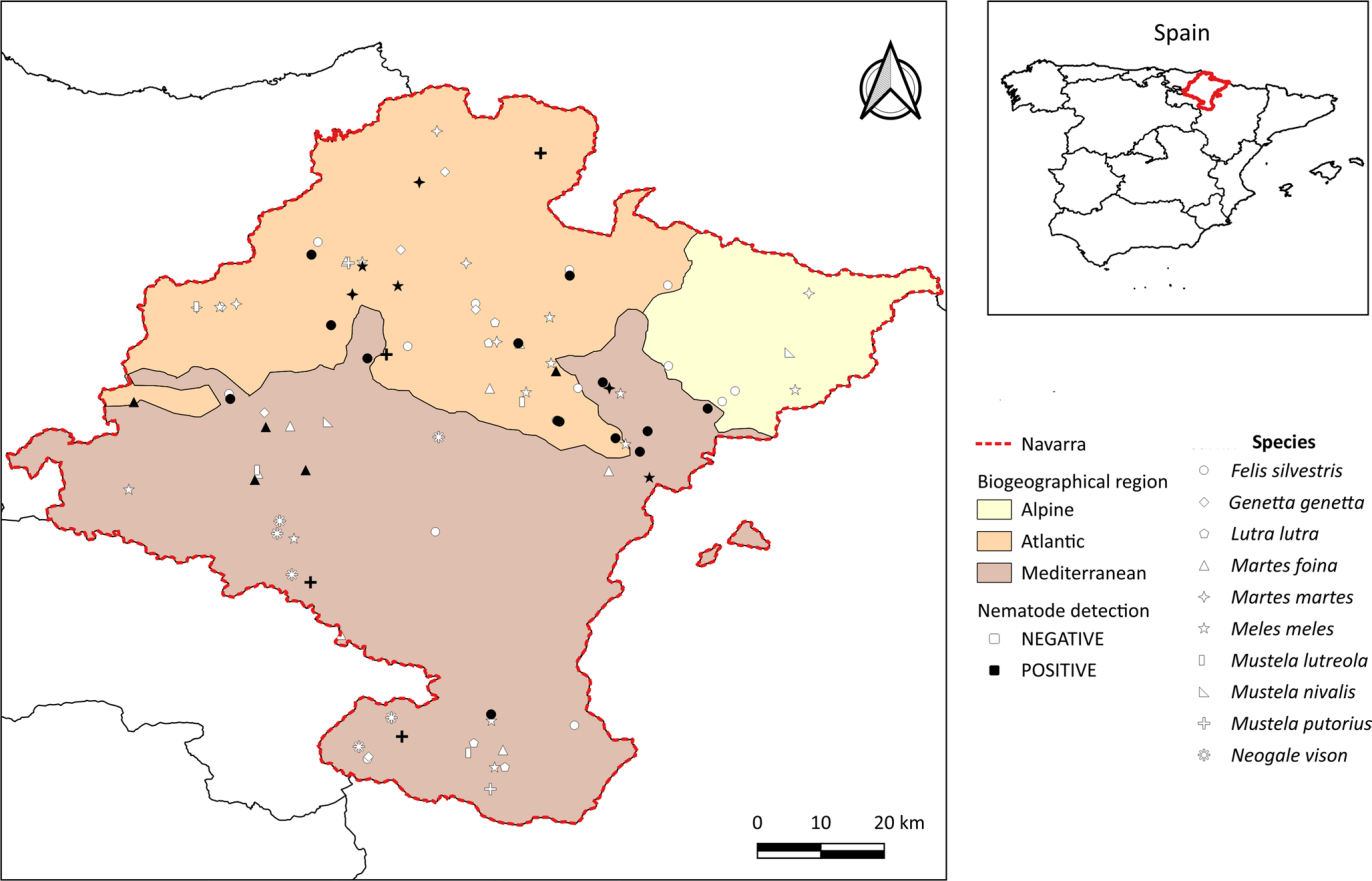
Map of Navarra showing the locations where the studied carcasses of wild carnivores were collected

No significant differences in CPN occurrence were observed among biogeographic areas (Fisher’s exact test, *p* > 0.05), with parasitized individuals detected throughout the region (Fig. 7). In wildcats, CPN occurrence was significantly higher in males (56.5%) than in females (20.0%) (*F* = 4.8, *p* < 0.05). A significant interaction between sex and age was also observed (*F* = 7.22, *p* < 0.05): prevalence in females was higher among adults, whereas in males it was higher among juveniles. No significant differences were found in the body condition index of wildcats in relation to parasitism status (*z* = 0.53, *p* > 0.05).

## Discussion

Although sample sizes were limited for several of the species included, it is important to recognize the logistical challenges associated with collecting road-killed carcasses of protected wild carnivores, which requires substantial coordination efforts. Despite these constraints, a notable number of European wildcats were successfully analyzed, providing the first data on this species in the Iberian Peninsula. The broad range of species examined enabled the detection of seven different cardiopulmonary nematode taxa, many of which represent novel host–parasite associations or new records for the Iberian Peninsula. As such, this study holds greater value for the qualitative faunistic information it provides rather than for its quantitative scope. Additionally, we report several novel genetic sequences for most of the recovered nematodes. These sequences will serve as valuable references for future research in parasitology, wildlife health, and disease ecology, facilitating the identification and comparison of nematodes across different host species and geographical regions.

The European wildcat is recognized as the natural host of *A. chabaudi*. Since its initial discovery in a wildcat from Greece (Diakou et al. 2016), the parasite has been reported in several other European countries (Gherman et al. 2016b; Giannelli et al. 2016; Veronesi et al. 2016; Stevanović et al. 2019). In Spain, *A. chabaudi* has only been documented in domestic cats from the Canary Islands (García-Livia et al. 2023). Therefore, our survey constitutes the first report of this parasite on the Iberian Peninsula and confirms the European wildcat as the primary reservoir maintaining the parasite in this region. Notably, phylogenetic analysis of both *cox1* and *ITS2* sequences placed our newly obtained sequences into a distinct clade, separate from other European *A. chabaudi* sequences, with strong bootstrap support. This finding suggests the existence of a potentially unique lineage in this area.

Research on cardiopulmonary nematodes in domestic cats from peninsular Spain remains extremely limited, with only two fecal-based studies conducted to date. Neither study detected the presence of *A. chabaudi* (Miró et al. 2004; Giannelli et al. 2017). However, the parasite’s presence in domestic cats from regions with stable wildcat populations, such as Navarre, cannot be ruled out and warrants further investigation.

*Angiostrongylus daskalovi* was first described in badgers from Bulgaria (Janchev and Genov 1988), but was largely forgotten until it was reported again by Gerrikagoitia et al. (2010) in the Basque Country, a region adjacent to Navarra. Gerrikagoitia et al. (2010) published a sequence from a recovered specimen; however, lacking reference material, they designated it as *Angiostrongylus* sp. The first molecular characterization of *A. daskalovi* was later performed on specimens from Romania (Gherman et al. 2016a), confirming that the specimen described by Gerrikagoitia et al. (2010) was indeed *A. daskalovi*. Thus, our study confirms the presence of this parasite in badgers from the Iberian Peninsula. Interestingly, *A. daskalovi* has also been found in invasive American minks from northwest Spain (Martínez-Rondán et al. 2017). To date, all documented cases in Iberia have been restricted to the humid, northern regions of Spain. This contrasts with observations by Nagy et al. (2021), who associated the presence of the parasite in badgers in Hungary with dry landscapes. These differences may be related to variations in the species of snails serving as intermediate hosts and their distinct ecological characteristics in Spain and Hungary. In this study, we contribute novel *ITS2* sequences as well as the first *cox1* sequences for *A. daskalovi*, expanding the molecular data available for this parasite.

*Troglostrongylus brevior* is a common component of the helminth fauna in European wildcats (Brianti et al. 2013; Falsone et al. 2014; Veronesi et al. 2016; Diakou et al. 2021, 2020; Bisterfeld et al. 2022). Until now, however, in Spain it had only been reported in domestic cats (García-Livia et al. 2023; Jefferies et al. 2010; Giannelli et al. 2017). The prevalence of *T. brevior* in wildcats in our study was lower than that reported in other studies on European wildcats, where prevalence rates between 30% and 70% have been documented (Falsone et al. 2014; Veronesi et al. 2016; Diakou et al. 2021; Bisterfeld et al. 2022). Nonetheless, our observed prevalence was higher than that reported in Romania (Deak et al. 2022) and in feral cats from the Canary Islands (García-Livia et al. 2023).

In Europe, *O. rostratus* has been detected in domestic cats from the Basque Country, Madrid, Mallorca, and the Canary Islands in Spain (Juste et al. 1992; Millán and Casanova 2009; Giannelli et al. 2017; García-Livia et al. 2023), as well as in Sardinia and Sicily in Italy (Brianti et al. 2014; Varcasia et al. 2015); and Hungary (Kiszely et al. 2019). In wildcats, *O. rostratus* had previously only been reported in Greece (Diakou et al. 2021). Therefore, our study represents the first record of *O. rostratus* in wildcats outside Greece, and the first report in the Autonomous Community of Navarre. Our findings align with those of Diakou et al. (2021), revealing a very low prevalence in the wildcat population, which may suggest that the wildcat is not a significant host in the epidemiology of this helminth.

As mentioned, *Crenosoma* spp. are common components of the helminth fauna in carnivores throughout Europe (Addison and Fraser 1994; Torres et al. 1996, 2001; Latrofa et al. 2015; Deak et al. 2023). In this study, *C. melesi* was confirmed in both badgers and polecats. The presence of *C. melesi* in polecats had previously been reported in a French region bordering Navarra (Torres et al. 2008) and in Catalonia (Torres et al. 2003). In Spain, this parasite has also been found in badgers, weasels (*Mustela nivalis*), and American minks (Feliu et al. 1996; Torres et al. 2001; Martínez-Rondán et al. 2017), indicating a broad host range. Phylogenetic analysis revealed that our sequences (one from a badger and another from a polecat) clustered with *C. melesi* sequences from Romanian badgers but formed a distinct branch separate from the main cluster. This divergence is unsurprising given the geographical distance between Romania and Spain, and suggests that badgers and polecats share the same parasite strains. Specimens from martens were classified as *C. petrowi*, though no readable sequences were obtained, likely due to the advanced decomposition of the carcasses that affected DNA quality. Nevertheless, *C. petrowi* is frequently reported as part of the helminth fauna in both marten species in Spain (Torres et al. 1996; Segovia et al. 2007).

*Eucoleus aerophilus* has been documented across Europe in both domestic animals (Traversa and Di Cesare 2013; Remesar et al. 2022; Samorek-Pieróg et al. 2024) and wildlife (Ribas et al. 2004; Dakova and Panayotova-Pencheva 2018; Schug et al. 2018), making its detection unsurprising given its wide distribution. In Spanish wildlife, it has been found in *Martes* spp. (Segovia et al. 2007; Torres et al. 1996) and canids (Gortázar et al. 1998; Segovia et al. 2004; Mañas et al. 2005; Martínez-Rondán et al. 2019). Because *E. aerophilus* has a direct life cycle (Anderson 2000), this likely explains its broad host range and distribution.

The remaining studied species did not show evidence of CPNs, although sample sizes were limited (ten or fewer individuals). Previous studies in genets (*Genetta genetta*) in Iberia similarly failed to detect CPNs (Feliu et al. 1996). However, American mink has been reported to host *A. daskalovi* and *C. melesi* in Galicia (Martínez-Rondán et al. 2017). Given its invasive nature, this species particularly warrants continued surveillance.

We also found differences in CPNs occurrence related to wildcat characteristics. The higher prevalence in males may be explained by their larger body size and greater home ranges, which likely increase prey consumption, or by immunosuppression effects associated with androgens (Schalk and Forbes 1997). The observation of higher prevaelnce in adult females compared to juvenile females could be related to increased cumulative exposure over time—although this pattern might also be expected in males—or may be secondary to the physiological costs of reproduction (Molina et al. 1999).

In summary, our study represents the first report of CPNs in carnivores from Navarre, as well as the first documented occurrence of these parasites in Iberian wildcats, thereby expanding current knowledge on their distribution across the Iberian Peninsula. We identified several novel host–parasite associations in the region, including, in some cases, the first records for this geographic area—such as *A. chabaudi*. Many of the parasites detected are shared among different wildlife species (*C. melesi* and *E. aerophilus*). Most notably, however, the CPNs found in wildcats are also shared with domestic cats, a widespread introduced species. Previous research on parasites in free-roaming domestic cats in Spain, both owned and feral, has documented high helminth burdens (Millán and Casanova 2009; García-Livia et al. 2023; Gassó et al. 2024). Moreover, Millán and Blasco-Costa (2012) demonstrated that nematode species can be transmitted between domestic cats and another wild feline, the Iberian lynx (*Lynx pardinus*). Whether similar interspecific transmission occurs with cardiopulmonary nematodes in our study area remains to be determined. Finally, given the subtle morphological differences among closely related nematode species and the often poor preservation of specimens collected from road-killed animals, we emphasize that molecular confirmation of species identification is highly recommended to ensure diagnostic accuracy.

## Data Availability

The sequence data obtained from this study have been deposited in the GenBank^®^database ([ncbi.nlm.nih.gov/genbank](https:/www.ncbi.nlm.nih.gov/genbank)). All sequences are availablein GenBank^®^ under the accession numbers: PV650321; PV650435; PV650643-45; PV651633-34; PV653661; PV653667; PV662153-57.
